# Protective effect of Dulaglutide, a GLP1 agonist, on acetic acid-induced ulcerative colitis in rats: involvement of GLP-1, TFF-3, and TGF-β/PI3K/NF-κB signaling pathway

**DOI:** 10.1007/s00210-024-03631-5

**Published:** 2024-11-23

**Authors:** Raghda N. El Mahdy, Manar A. Nader, Manar G. Helal, Sally E. Abu-Risha, Marwa E. Abdelmageed

**Affiliations:** 1 Department of Pharmacology and Toxicology, Faculty of Pharmacy, Mansoura University, Mansoura, 35516 Egypt; 2 Department of Pharmacology and Toxicology, Faculty of Pharmacy, Tanta University, Tanta, Egypt; 3 Department of Pharmacy Practice, Faculty of Pharmacy, Sinai University- Kantra Branch, Ismailia, Egypt

**Keywords:** Ulcerative colitis, Dulaglutide, Protein kinase B, Phosphatidylinositol-3-kinase, Trefoil factor-3, Transforming growth factor-1

## Abstract

A chronic inflammatory condition of the colon called ulcerative colitis (UC) is characterized by mucosal surface irritation that extends from the rectum to the near proximal colon portions. The rationale of this work was to conclude if dulaglutide (Dula) could protect rats from developing colitis caused by exposure to acetic acid (AA). Rats were randomly divided into seven groups (each with eight rats): Normal control, Dula control, AA (received 2 milliliters of 3% v/v AA through the rectum), Sulfasalazine (SLZ); given SLZ (100 mg/kg) orally from day 11 to day 21 then AA intrarectally on day 22 and Dula groups ( pretreated with 50, 100 or 150 μg/kg subcutaneous injection of Dula - once weekly for three weeks and AA on day 22 to induce ulcerative colitis, colon tissues and blood samples were taken on day 23. By generating colonic histological deviations such as inflammatory processes, goblet cell death, glandular hyperplasia, and mucosa ulcers, Dula dropped AA-induced colitis. Additionally, these modifications diminished blood lactate dehydrogenase (LDH), C-reactive protein (CRP), colon weight, and the weight/length ratio of the colon. In addition, Dula decreased the oxidative stress biomarker malondialdehyde (MDA) and increased the antioxidant enzymes (total antioxidant capacity (TAC), reduced glutathione (GSH), and superoxide dismutase (SOD) concentrations). Dula also significantly reduced the expression of transforming growth factor-1 (TGF-β1), phosphatidylinositol-3-kinase (PI3K), protein kinase B (AKT) signaling pathway, and the inflammatory cytokines: nuclear factor kappa B (NF-κB), interleukin-6 (IL-6), and interferon-γ (IFN-γ) in colonic cellular structures. In addition, Dula enforced the levels of glucagon-like peptide-1 (GLP-1) and trefoil factor-3 (TFF-3) that were crucial to intestinal mucosa regeneration and healing of wounds. By modulating TGF-β1 in conjunction with other inflammatory pathways like PI3K/AKT and NF-κB, regulating the oxidant/antioxidant balance, and improving the integrity of the intestinal barrier, Dula prevented AA-induced colitis in rats.

## Introduction

Inflammatory bowel disorders (IBDs) are persistent, reoccurring intestinal inflammatory syndromes with complicated and unclear pathophysiology, including genetic, immunological, and environmental factors (Zatorski et al. 2019). IBD may manifest as Crohn’s disease (CD) and ulcerative colitis (UC). The chief characteristic of UC, a chronic inflammatory colon disease, is continued mucosal surface inflammation extending from the rectum to the proximal colon region (Binabaj et al. 2019). Clinical symptoms common to patients with UC include fever, tiredness, weight loss, and bloody diarrhea, which is another common symptom (Boal Carvalho and Cotter 2017). Although the precise cause of UC is unknown, various mechanisms, including neutrophil infiltration, high concentrations of cytokines, and reactive oxygen compounds (ROS) which promote inflammation, are designated to play a role in the pathophysiology of UC (Balmus et al. 2016). Most UC therapies now available target patients’ inflammatory and immunological responses (Ilan 2016). In IBD therapy, 5-aminosalicylates, immunosuppressive drugs, corticosteroids, and biological therapies are often used; nevertheless, some patients do not react to treatment; thus, it is crucial to take into account the detrimental adverse effects and cost of managing people with IBD (van der Valk et al. 2016). These observations suggest that potential therapies for resistant and recurring UC illnesses include medications that imitate the normal course of wound healing and resolution.

Intrarectal instillation of AA is followed by protonation and migration of molecule of AA into colonic microflora, resulting in destruction of colonic epithelium, increased bacterial influx into the lamina propria, and enhancement of the activated enterocytes to generate a variety of cytokines (Soliman et al. 2019). Therefore, AA is a powerful inducer of inflammation with the production of pro-inflammatory cytokines such as interleukin-6 (IL-6) (Ghasemi-Pirbaluti et al. 2017) as well as nuclear factor kappa B (NF-κB) and interferon-γ (IFN-γ) (El-Akabawy and El-Sherif 2019b) which ultimately result in ulceration and damage of the mucosa. In addition, it increases the level of malondialdehyde (MDA) (an indicator of lipid peroxidation) and reduces the level of both reduced glutathione (GSH) and superoxide dismutase (SOD) in colonic mucosa, resulting in the disruption of the oxidant/antioxidant system and damage of the colonic mucosa (Cetinkaya et al. 2005). Furthermore, AA provokes a loss of balance between cell proliferation and death leading to the loss of tissue homeostasis and apoptosis (Ali et al. 2017).

In UC, a disrupted intestinal barrier leads to the translocation of the gastrointestinal microbes into the intestinal tissue resulting in inflammation (Michielan and D'Incà, 2015). The peptide hormone glucagon-like peptide-1 (GLP-1), which is secreted by intestinal endocrine L cells, regulate metabolic processes (Yaribeygi et al. 2021) as GLP-1 is implicated in pathophysiological processes that can lead to inflammation, cardiovascular, and nervous system disorders (Lee and Jun 2016, Rakipovski et al. 2018, Li et al. 2021). Curiously, GLP-1 inhibits the NF-κB signaling pathway and alleviates acute lung injury in lipopolysaccharide (LPS)-induced mice (Xu et al. 2019). Wang et al. showed that GLP-1 analog liraglutide reduced inflammatory cytokine expression in rat hippocampus and prevented neuroinflammation (Wang et al. 2018). Furthermore, the distal colon and ileum are home to enteroendocrine cells that predominantly manufacture GLP-1, and it is crucial for controlling blood sugar levels (Wu et al. 2021). Numerous physiological effects of GLP-1 include increased satiety, delayed stomach emptying, and weight loss (Andersen et al. 2018). GLP-1 has a very brief half-life (less than 2 min) owing to the swift deactivation of the commonly proteolytic enzyme dipeptidyl peptidase-4 (DPP-4), which leads to the emergence of GLP-1 receptor agonists (GLP-1RAs), which are GLP-1 analogs with extended half-lives and ongoing biological activity (Ma et al. 2021). GLP-1RAs have been shown in several studies to have curative and preventive advantages in a wide range of systems, involving vascular protection, neuroprotection, anti-inflammatory, and anti-fibrosis activities, alongside their relevance in controlling diabetes (Wu et al. 2021).

Dula is a long-acting GLP-1 RA with 90% human homology that was approved by the US FDA in 2014 (half-life: 5 days). According to research, Dula reduces patients’ levels of HbA1c, a marker of type 2 diabetes, and is superior to liraglutide in terms of weight reduction (Dungan et al. 2014). Long-term use of Dula decreases fasting and fed blood glucose levels, promotes islet beta cell activity, and promotes the release of blood glucose-dependent insulin (Dagenais et al. 2020). Dula has recently been found to have a strong anti-inflammatory effect on rats’ disuse muscle atrophy and synoviocytes that resemble fibroblasts (Zheng et al. 2019, Nguyen et al. 2020).

To develop novel approaches and concepts for the therapeutic management of UC, this investigation pointed to exploring the mechanism underlying the action of the GLP-1RA, Dula, on experimentally generated colitis in rats. Therefore, this study aimed to examine the possible beneficial effect of Dula against AA-induced UC and to elucidate the underlying mechanisms.

## Materials and methods

### Experimental animals

Fifty-six male Sprague-Dawley rats weighing 200 ± 20 g each an average age of 6–8 weeks were consumed from MERCK factory in Mansoura, Egypt. They were kept at constant environmental conditions throughout the experimental period with a room temperature ranging from 20 to 26°C with regular light cycles of 12/12–10/14 h light/dark, relative humidity at the level of rat cages of 40–70% with room ventilation rates of about 15–20 air changes/h. Rats were allowed free access to food and water and were left to acclimatize for one week before commencing the experimental protocol. The animals were housed 4 per cage. Rats had a 24-h fast before AA-induced colitis introduction. During the trial, regular food and water were allowed. The Mansoura University Faculty of Pharmacy’s Committee of Scientific Research Ethics approved the field experiment used in this study; the code (2023-122) was derived from the Ph.D. thesis code (2021-274) where all the procedures such as anesthesia, analgesia, and euthanasia protocols were followed according to the guidelines. Throughout the investigation, the National Institutes of Health Guide for the Care and Use of Laboratory Animals (NIH publication no. 85-23, revised 2011) was obeyed.

### Chemicals and drugs

Dula was available as an injection-ready solution under the brand name TRULICITY from Lilly USA, LLC, US.

Tablet varieties of the drug sulfasalazine (SLZ) were provided (Salazosulphpyrine^EN^, Kahira Pharmaceuticals & Chemical Industries Co., Victoria Sq., Shoubra, Cairo, Egypt). Rats received the oral gavage after the pills were broken and the appropriate dosages had been diluted in distilled water. For the induction of UC, from the El-Gomhoria chemical business in Mansoura, Egypt, AA was bought. All other compounds are put through the most stringent analytic grading.

### Rats with colitis are induced

In advance of obtaining anesthesia with ether inhalation, rats were starved for 24 h. The rats received diluted AA (2 mL, 3% v/v) over 30 s using a 2-mm diameter polyurethane cannula placed 8 cm into the rat’s anus, and the volume and concentration of AA administered were standardized and reproducible to maintain consistency in the experimental protocol.

To stop AA leaking, the animals were held upside down for two minutes. Using the same approach, 2 ml of 0.9% NaCl was administered to normal rats in place of AA, and the safety and well-being of the animals were evaluated to ensure the safety and well-being of the animals during the experimental procedure according to the literature (Babitha et al. 2019, Elshazly et al. 2020, El Mahdy et al. 2023).

### Experimental design

**Fig. 1:**
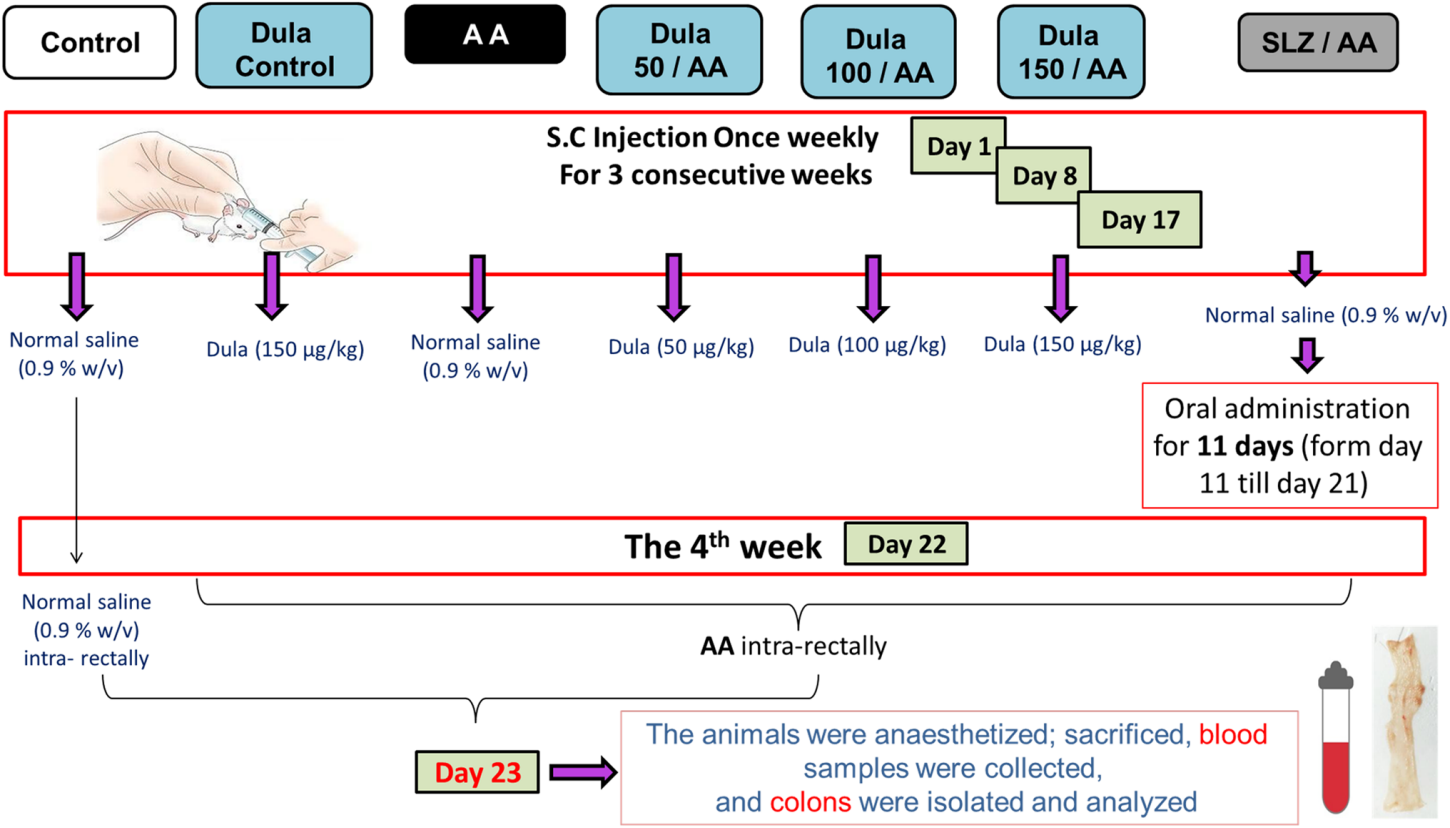
Schematic presentation of the experimental design. AA: acetic acid; SLZ: sulfasalazine; Dula: dulaglutide

Animals were allocated at random into seven test groups, each with eight rats (Fig. 1):

1. Rats in the normal control group got normal saline (0.9 % w/v, subcutaneously (s.c.)) once weekly for three weeks straight; then on the 22^nd^ day of the study, they got a 0.9 % w/v of normal saline administration into the colon.
2. The Dula control group served as the drug control group; rats were given Dula (150 μg/kg, s.c.) once weekly for three weeks straight; then on the 22^nd^ day of the study, they obtained a normal saline instillation (0.9% w/v) into their colons (Wu et al. 2021).
3. The AA group served as the diseased group; following the procedure of receiving normal saline (0.9% w/v, s.c.) once per week for three consecutive weeks, rats obtained injection through the rectum of AA on the 22^nd^ day of the study (Babitha et al. 2019, Elshazly et al. 2020, El Mahdy et al. 2023).
4. In the SLZ + AA group, rats were used as the standard group; during the first three weeks, they were given 0.9% NaCl s.c. once a week. Subsequently, they were given SLZ (100 mg/kg) orally for 11 days (from day 11 to day 21) then AA intrarectally on day 22 (Babitha et al. 2019, Elshazly et al. 2020).
5. The Dula 50 μg/kg + AA group served as the treatment group; this group of rats got Dula (50 μg/kg, s.c.) once per week for three weeks in succession. On the fourth week (day 22), AA was added intrarectally (Wu et al. 2021).
6. The Dula 100 μg/kg + AA group served as the treatment group, and rats in this group got AA intrarectally on the 22^nd^ day of the fourth week after receiving Dula (100 μg/kg, s.c.) for three weeks in a row (Vallöf et al. 2020).
7. The Dula 150 μg/kg + AA group served as the treatment group; this group of rats was treated with Dula (150 μg/kg, s.c.) once every week for three consecutive weeks, then, on the fourth week, intrarectal administration of AA (day 22) (Wu et al. 2021). The doses of Dula were selected based on earlier studies (Vallöf et al. 2020, Wu et al. 2021).

Rats were given thiopental sodium (40 mg/kg by intra-peritoneal injection) to make them unconscious 24 h following the administration of AA; their vital signs were monitored during anesthesia as rectal temperature, heart rate, and respiratory rate. A retro-orbital puncture was used to obtain blood, and serum was detached to clarify the biological process. Then, the animals were sacrificed, and the abdomen was opened longitudinally with a midline incision that extended to the rectum. The rectum was removed from the distal part, and then the last 8 cm of the rats’ colons (measured with a ruler) were removed, longitudinally opened, washed softly with 0.9% NaCl and dried with tissue. Following this procedure, colon weight was assessed, and macroscopic scoring was carried out. Similar divisions were made into three pieces using the gathered colons, the first one, located approximately 1 cm from the distal colon, was embedded in 10% formalin and preserved in paraffin wax for histological inspections and immunohistochemical investigation, and the second, homogenized in phosphate-buffered saline (PBS; 10% w/v, pH 7.4) using Omni-125 hand-held homogenizer (Omni International, USA), then the homogenates were centrifuged using a cooling centrifuge at (2000× *g*, 15 min, 4 °C), and the supernatant was divided into aliquots, where one aliquot was freshly used for assay of oxidative biomarkers, and the other one stored at −80 °C to be used for enzyme-linked immunosorbent assay (ELISA). The third part was flash-frozen in liquid nitrogen and stored at −80 °C.

### Estimate of the weight/length ratio and *colon* weight

We measured the weight and length of each rat’s separated colon and computed weight-to-length ratios.

### Estimating the macroscopically colonic score

According to the findings of Mei et al. (Mei et al. 2005), the macroscopic inflammatory scores of the colon were evaluated. Using a scale from 0 to 4, the following parameters were applied to measure macroscopic harm based on clinical signs and standardized to all animals in the study: 0, the absence of macroscopic abnormalities; 1, mucosal erythema; 2, small bleeding or minute erosions; 3, considerable edema; and 4, severe ulceration, edema, and tissue necrosis.

### Serum total antioxidant capacity (TAC), lactate dehydrogenase (LDH) activity, and C-reactive protein (CRP) level measurement

Serum activity of LDH and CRP level were measured biochemically using colorimetric kits using Human Diagnostics kit (Cat no. 12214, Wiesbaden, Germany) and Spinreact kit (Cat no. SP110700, Barcelona, Spain), respectively, in compliance with established standards. The serum TAC was ascertained using a kit (Cat no. TA2513, Biodiagnostic, Giza, Egypt).

### Quantification of colonic redox balance biomarkers

MDA, GSH, and SOD levels had been assessed for colon homogenates using the techniques outlined in (Guesdon et al. 1979, Ohkawa et al. 1979, El-Akabawy and El-Sherif 2019a) respectively.

For MDA assessment, thiobarbituric acid reactive substances were assessed. Briefly, 0.2 mL of colon homogenate was mixed with 0.2 mL of 8.1% sodium dodecyl sulfate, 1.5 mL of 20% acetic acid solution adjusted to pH 3.5 with NaOH, and 1.5 mL of 0.8% aqueous solution of TBA. The reaction was incubated at 95 °C for 60 min followed by centrifugation at 2000× g for 10 min. The absorbance of the organic layer was measured at 532 nm.

In addition, non-protein sulfhydryl compounds were estimated by utilizing trichloro acetic acid-deproteinized tissue supernatant for determination of GSH. Briefly, 0.5mL of 50% (*w*/*v*) trichloro acetic acid was used to precipitate the protein in colon homogenate. The supernatant was collected after centrifugation at 1000× *g* for 5 min, and 0.1 mL of the supernatant was mixed with 1.7 mL phosphate buffer (0.1 M, pH 8) and 0.1 mL Ellman’s reagent. The reaction was kept at room temperature for 5 min. Yellow was formed and measured spectrophotometrically at 412 nm.

For measuring SOD activity, the degree of inhibition of pyrogallol autooxidation by SOD enzyme at alkaline pH was used. The change of absorbance at 420 nm per minute was monitored over 3 min. Controls with no samples were run under the same conditions to compute the rate of inhibition. The enzyme activity was expressed as U/mg where one unit represents the number of enzymes that suppresses pyrogallol autooxidation by 50%.

### Tissue IL-6 and NF-κB expression using immunohistochemical (IHC) analysis

The protein expression of IL-6 and NF-κB in the colonic tissues was determined using immunohistochemistry technique utilizing polyclonal antibodies (Cat nos. PA5–27617 and PA1–26811, respectively; Thermo Fisher Scientific Anatomical Pathology 46360 Fremont Blvd., CA 94538, USA) according to the Biotin-Avidin complex technique (Guesdon et al. 1979). The colonic sections with 4 µm thickness were deparaffinized followed by immersion in H_2_O_2_ to stop the activity of endogenous peroxidase. Sections were boiled in citrated buffer (0.01 mol/L) to restore antigen, followed by the addition of blocking solution (5% BSA) and incubation for 20 min at 37°C. The colonic sections were incubated with the antibodies for 12 hours at 4 °C, followed by incubation with biotinylated goat anti-rabbit IgG at 25 °C for 20 min. SABC reagent was added to colonic sections and incubated at 26 °C for 20 min, followed by a 3,3-diaminobenzidine addition. Sections were examined using Olympus, Tokyo, Japan, and optical densities were determined using the Image-Pro Plus program. Consistent with the previous studies, the locations and abundances of NF-κB and IL-6 colonic expression levels have been identified (Wang et al. 2010, El-Akabawy and El-Sherif 2019a).

### Estimation of the colonic levels of GLP-1, trefoil factor-3 (TFF-3), transforming growth factor-1 (TGF-β1), phosphatidylinositol-3-kinase (PI3K), protein kinase B (AKT), and IFN-γ using ELISA technique

The levels of GLP-1, TFF-3, and TGF-β1 were determined by the instructions provided by freely available ELISA kits: GLP-1 (Cat no. E-EL-R3007, Elabscience, Texas, USA), TGF-β1 (Cat no. SCA124Ra, Cloud-Clone Corp., Texas, USA), and TFF-3 (Cat no. ER0574, Fine test, Wuhan Fine Biotech Co., Wuhan, China).

Additionally, PI3K, AKT, and IFN-γ were determined by the instructions provided by freely available spectrophotometric kits: PI3K (Cat no. orb411021, Biorbyt Ltd., Cambridge, United Kingdom), AKT (Cat no. RTFI01064, Assay Genie, Dublin 2, Ireland), and IFN-γ (Cat no. E0103Ra, Bioassay technology laboratory, Shanghai, China) according to the given instructions.

### Estimated microscopic score and changes in colonic histopathology

Hematoxylin and eosin (H&E) staining of 5 µm cuts of the colon preserved in paraffin wax has been used to assess the degree of colonic damage and histological changes and the measurement and evaluation of mucosal edema, necrosis, inflammation, and ulceration severity.

### Statistical analysis

Results underwent either median ± interquartile range (IQR) or mean ± standard error (SEM) calculations. To determine whether the data was normal, the Kolmogorov-Smirnov test was used. One-way analysis of variance (ANOVA) and Tukey’s post hoc test were used for parametric results; the Kruskal-Wallis test and Dunn was used for non-parametric results. Statistical significance was established at *p* <0.05. For the statistical evaluation, GraphPad Prism V 5.02 (GraphPad Software Inc., San Diego, CA, USA) was used.

## Results

### Effect of Dula (50, 100, and 150 μg/kg) on macroscopic scores

**Fig. 2:**
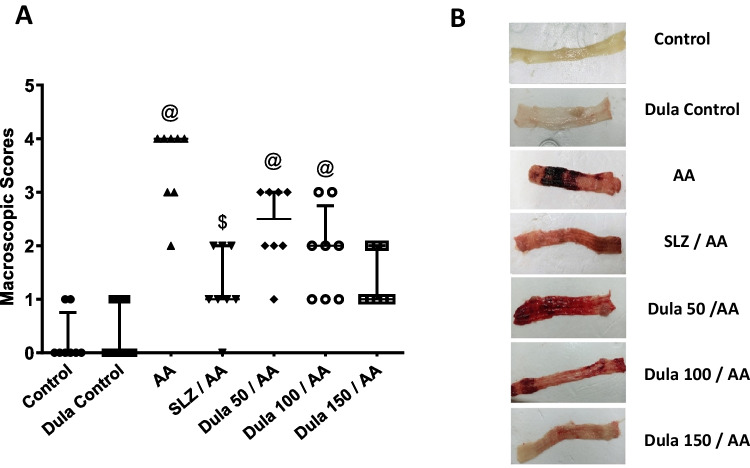
Effect of Dula (50, 100, and 150 μg/kg) on macroscopic scores. **A** Macroscopic scores and **B** photographed colon tissues. Data were expressed as median ± IQR. AA: acetic acid; SLZ: sulfasalazine; Dula: dulaglutide. ^@,$^*p*<0.05, significantly different from the control and AA-injected groups, respectively, employing Tukey-Kramer multiple comparisons post hoc test after one-way ANOVA

Opposed to the colons of the control group (Fig. 2) that exhibited typical features, the colons of the rats given AA had noteworthy erythema, thickness, necrosis, and edema. However, when SLZ and Dula (100 and 150 μg/kg) were administered, the modifications were less pronounced in the colons of the AA-treated group. Dula (50 μg/kg) showed a slight effect compared to the other doses. This result showed that Dula in high doses could be effective in AA-induced colitis treatment.

### Colon weight and weight/length ratio of Dula (50, 100, and 150 μg/kg)

Table 1 confirms that AA inoculation into the rectum augmented wet weight and weight/length by 1.40 times (*p* < 0.05) in comparison to the control group. Compared to the AA group, SLZ pretreatment animals experienced a significant (*p* < 0.05) fall in these indicators (by 22.95%). Rats getting 50, 100, and 150 μg/kg of Dula pretreatment revealed considerable (*p* < 0.05) drops in each parameter contrasted to the AA-treated group, with falls of 5.4%, 16.3%, and 21.06%, correspondingly.

**Table 1:** Effect of Dula (50, 100, and 150 μg/kg) on AA-induced changes on colon weight and colon weight/length ratio of rats

Parameters	Groups						
	Control	Dula control	AA	SLZ+AA	Dula 50 μg/kg + AA	Dula 100 μg/kg + AA	Dula 150 μg/kg + AA
Colon weight (g)	0.660 ± 0.03698	0.4800 ± 0.02619	0.9265 ± 0.04329^$^	0.7138 ± 0.05130^λ^	0.8763 ± 0.05444^$^	0.7750 ± 0.03973	0.7313 ± 0.03861^λ^
Colon weight/length ratio (mg/cm)	82.50 ± 4.623	60.00 ± 3.273	115.8 ± 5.412^$^	89.22 ± 6.413^λ^	109.5 ±6.805^λ^	96.88 ±4.966^λ^	91.41 ± 4.826^λ^

Data were expressed as mean ± SEM (*n* = 8)

*Dula* dulaglutide, *AA* acetic acid, *SLZ* sulfasalazine

^$,λ,ϕ,∇^*p*<0.05, significantly different from the control group, AA-injected group, SLZ-treated group, and Dula 50 μg/kg-injected group, respectively, using one-way ANOVA followed by Tukey-Kramer multiple comparisons post hoc test

### The effectiveness of Dula at 50, 100, and 150 μg/kg on the colon’s histological alterations

**Fig. 3:**
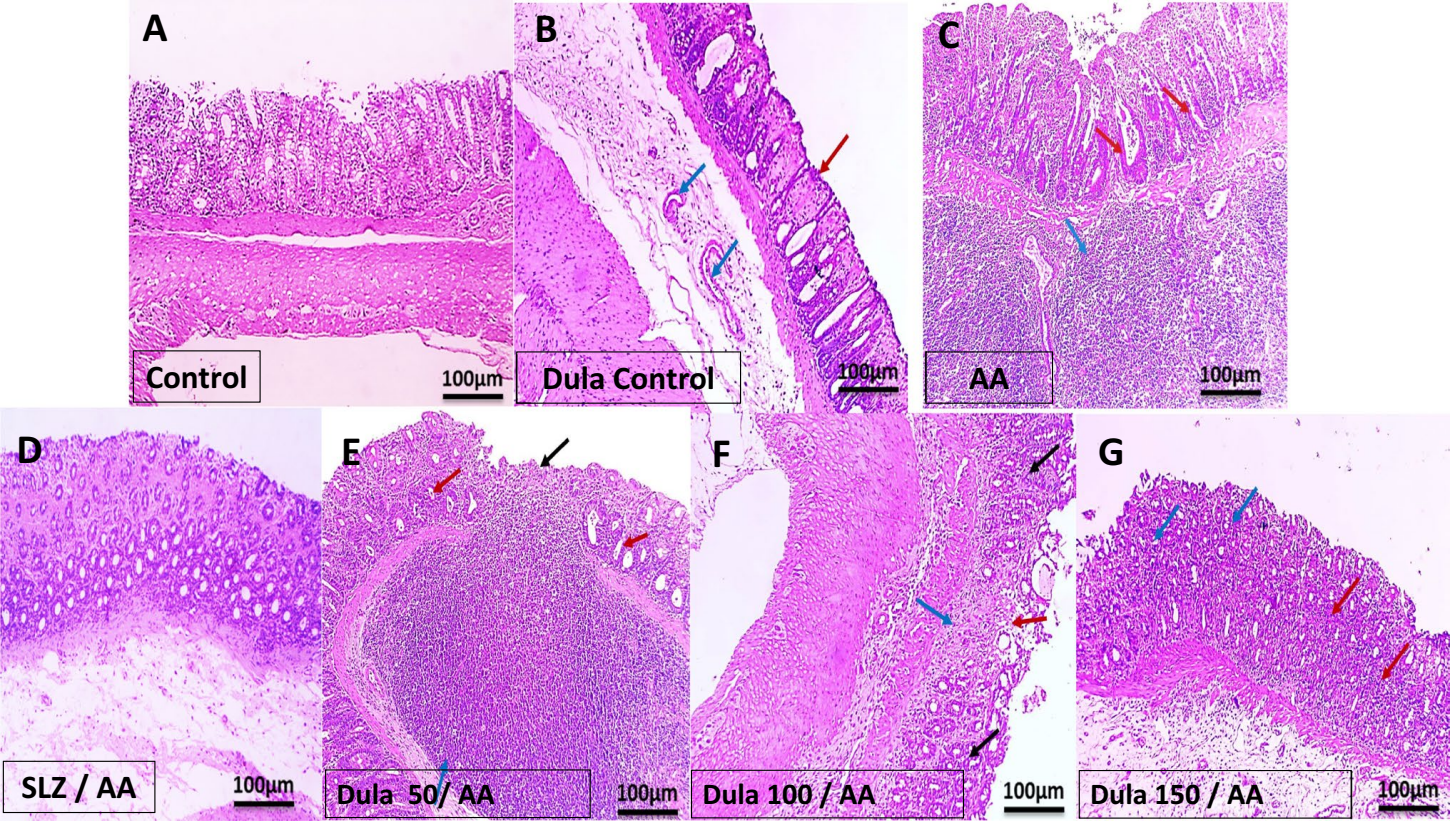
Effect of Dula (50, 100, and 150 μg/kg) on colon histopathological changes (H&E Bar size: 100 μm). **A** Sections of the normal colon showed normal colonic mucosal glands, average in size and shape lined with columnar cells and goblet cells separated by fibroblastic stroma. **B** Section of colon with only Dula 150 μg/kg protection (control) average-sized colonic mucosa (red arrow) with underlying submucosa with mild congested vessels (blue arrows). **C** Section of the diseased colon (AA) showed sub-mucosal large infiltrate of chronic inflammatory cells with lymphoid follicle formation score 3 (blue arrow) with hyperplasia of glands score 2 and loss of goblet cells score 2 (red arrows). **D** Section of the colon SLZ-treated group showed individual chronic inflammatory cells infiltrate score 1 with hyperplastic glands score 1 with no loss of goblet cells score 0. **E** Section of the colon of Dula 50 μg/kg-treated group showed focal ulceration score 3 (black arrow) with heavy inflammation score 3 (blue arrow), glandular hyperplasia score 2 with goblet cells loss score 2 (red arrow). **F** Section of the colon of Dula 100 μg/kg-treated group showed healed ulcer (red arrow) with mild inflammation score 2 (blue arrow), glandular hyperplasia score of 1 with goblet cells loss score 1 (black arrows). **G** Section of colon of Dula 150 μg/kg-treated group showed no ulceration with individual cell infiltration score 1 (red arrows), moderate glandular hyperplasia score 2 (blue arrows) with no goblet cells loss score 0

Figure 3 illustrates normal colonic mucosal glands that are average in size and form and surrounded by goblet and columnar cells of H&E-dyed colon layers from the Dula control and normal colon which showed a fibroblastic matrix that had been split into smaller sections by individual fibroblasts (Fig. 3A, B). Colonic sections from the AA-injected group, on the other hand, revealed a sub-mucosal massive infiltration of chronic inflammatory cells with the development of lymphoid follicles, gland hyperplasia, as well as goblet cell death (Fig. 3C).

Rats pretreated with 50, 100, and 150 μg/kg of Dula and SLZ reported distinct instances of chronic inflamed cell infiltration with hyperplastic glands, but not the disappearance of goblet cells in colon sections (Fig. 3D), indicating a considerable reduction in the histological harm caused by AA. Sections of the colon from the Dula 50 μg/kg group revealed glandular hyperplasia with loss of goblet cells, localized ulceration, and significant inflammatory response (Fig. 3E). Sections of the colon from the Dula 100 μg/kg group showed glandular hyperplasia, a healed ulcer with minimal inflammation, and a decrease in goblet cells (Fig. 3F). A substantial quantity of glandular hyperplasia without goblet cells dying and no ulceration with individual cell invasion appeared in sections of the colon from the Dula 150 μg/kg-treated group (Fig. 3G). Interestingly, compared to the lower dosages (50 and 100 μg/kg) of Dula was greater than the greater one (150 μg/kg).

**Fig. 4:**
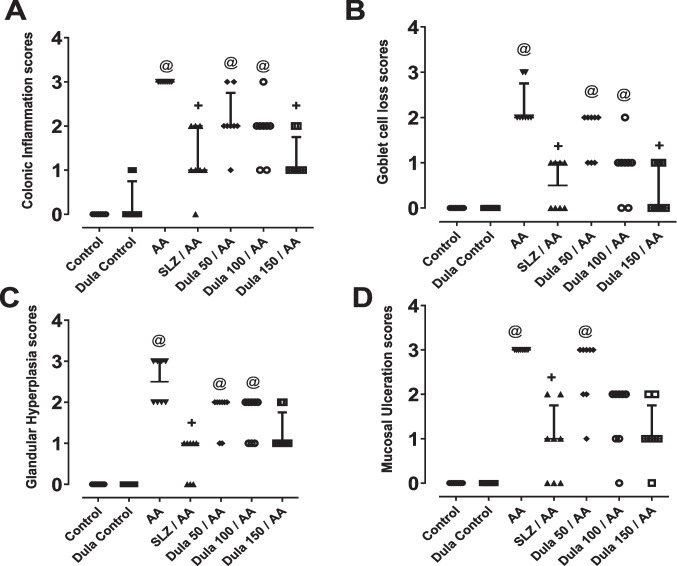
Histopathology-based scatter dot plots of mucosal ulceration grades. **A** colonic inflammation; **B** goblet cell loss; **C** glandular hyperplasia; **D** ulceration. Data were expressed as median ± IQR. ^@, +^*p*<0.05, significantly different from the control and AA-injected groups, respectively, applying the multiple comparison test of Dunn after the Kruskal-Wallis test

As seen in (Fig. 4), rats treated with AA had much higher inflammation score 3 (Fig. 4A), Contrary to the control group, these animals developed goblet cell depletion score 2 (Fig. 4B), glandular hyperplasia score 2 (Fig. 4C), and more mucosal ulceration score 3 (Fig. 4D). In contrast to the AA group, treatment with Dula (50, 100, and 150 μg/kg) with the traditional SLZ caused a substantial decrease in all the preceding values.

### Effectiveness of Dula (50, 100, and 150 μg/kg) on oxidant/antioxidant: TAC, MDA, GSH, and SOD

Table 2 shows that relative to the control group, AA administration noteworthy (*p* < 0.05) decreased the serum levels of serum TAC, colonic GSH, and SOD levels by 51.33%, 77.46%, and 49.16%, respectively. Additionally, MDA concentration was 4.39 times higher in AA-induced colitis animals than in control rats (*p* < 0.05). In comparison with the AA group, the traditional SLZ substantially (p < 0.05) boosted these values by 1.94, 3.10, and 1.69 times, correspondingly. Additionally, it led to a 67.97% large drop in MDA levels.

**Table 2:** Impact of Dula (50, 100, and 150 μg/kg) on oxidative stress biomarkers (TAC, GSH, SOD, and MDA)

Parameters	Groups						
	Control	Dula control	AA	SLZ+AA	Dula 50 μg/kg + AA	Dula 100 μg/kg + AA	Dula 150 μg/kg + AA
TAC	0.7038 ± 0.03586	0.7165 ± 0.02442	0.3425 ± 0.03312^$^	0.6677 ± 0.03928^λ^	0.5010 ± 0.03562^$λϕ^	0.5944 ± 0.02876^λ^	0.6829 ± 0.02002^λ∇^
GSH	4.487 ± 0.1466	4.092 ± 0.1073	1.011 ± 0.1022^$^	3.141 ± 0.2640^$λ^	1.688 ± 0.1445^$ϕ^	2.248 ± 0.2036^$λϕ^	2.881 ± 0.2421^$λ∇^
SOD	492.6 ± 14.82	445.8 ± 5.121	250.4 ± 17.58^$^	424.3 ± 14.45^λ^	317.2 ± 23.02^$ϕ^	352.8 ± 18.15^$λϕ^	417.0 ± 15.34^$λ∇^
MDA	69.31 ± 4.532	70.70 ± 4.638	304.8 ± 19.22^$^	97.61 ± 5.359^λ^	177.7 ± 15.64^$λϕ^	146.9 ± 12.45^$λ^	115.6 ± 9.130^λ∇^

Data were expressed as mean ± SEM (*n* = 8)

*Dula* dulaglutide, *AA* acetic acid, *SLZ* sulfasalazine, *TAC* total antioxidant capacity, *MDA* malondialdehyde, *GSH* reduced glutathione, *SOD* superoxide dismutase

^$,λ,ϕ,∇^*p*<0.05, significantly different from the control group, AA-injected group, SLZ-treated group, and Dula 50 μg/kg-injected group, respectively, using one-way ANOVA followed by Tukey-Kramer multiple comparisons post hoc test

Contrary to the AA group, medication with Dula (50 μg/kg) significantly (*p* < 0.05) elevated TAC, colonic GSH, and SOD levels by 1.46, 1.66, and 1.26 times, respectively. Rats given Dula (100 μg/kg) showed significantly higher blood TAC, colonic GSH, and SOD concentrations (1.73, 2.22, and 1.40 times, respectively) than those given AA (*p* < 0.05). In the group Dula (150 μg/kg), serum TAC, colonic GSH, and SOD levels noticeably (*p* < 0.05) raised by 1.99, 2.84, and 1.66 times, respectively. Treatment with Dula (50, 100, and 150 μg/kg) significantly (*p* < 0.05) decreased colonic MDA levels by 41.69%, 51.80%, and 62.07%, respectively, as matched to the AA group. These outcomes revealed that Dula exhibited antioxidant action and was more effective at high doses (150 μg/kg) than at lower doses (50 and 100 μg/kg).

### Effectiveness of Dula (50, 100, and 150 μg/kg) on colonic GLP-1 expression levels

**Fig. 5:**
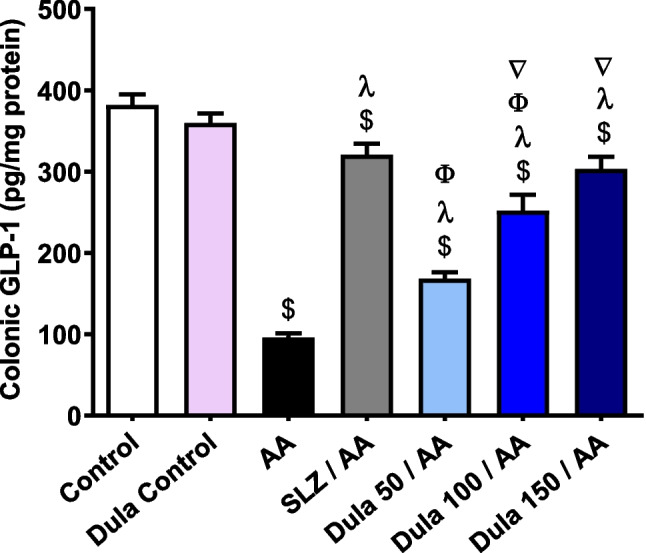
Effect of Dula (50, 100, and 150 μg/kg) on colonic levels of GLP-1. Data were expressed as mean ± SEM (*n* = 8). AA: acetic acid; SLZ: sulfasalazine; Dula: dulaglutide; GLP-1: glucagon like peptide-1. Data were expressed as mean ± SEM (*n* = 8). ^$,λ,ϕ,∇^*p*<0.05, significantly different from the control group, AA-injected group, SLZ-treated group, and Dula 50 μg/kg-injected group, respectively, employing Tukey-Kramer multiple comparisons post hoc test after one-way ANOVA

AA installation led to a significant (*p* < 0.05) fall in colonic GLP-1 levels of 74.91% versus control rats (Fig. 5). Contrary to the AA group, traditional SLZ indicated a worrisome (*p* < 0.05) rise of this parameter by a factor of 3.34-fold. Applying Dula at dosages of 50, 100, and 150 μg/kg markedly (*p* < 0.05) elevated the lowered quantity of GLP-1 by 1.75-, 2.63-, and 3.16-folds, respectively, versus the group that received AA. Higher doses from Dula (100 and 150 μg/kg) markedly elevated GLP-1 levels in comparison to the lower dosage (50 μg/kg), although Dula (150 μg/kg) did not statistically vary from the SLZ group.

### Efficacy of Dula (50, 100, and 150 μg/kg) on the colonic expression level of TFF-3

**Fig. 6:**
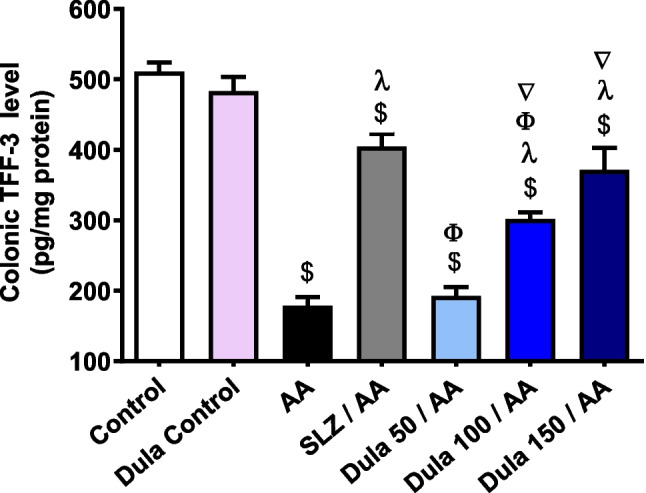
Effect of Dula (50, 100, and 150 μg/kg) on the colonic expression level of TFF-3. Data are expressed as mean ± SEM (*n* = 8). AA: acetic acid; SLZ: sulfasalazine; Dula: dulaglutide; TFF-3: trefoil factor-3. ^$,λ,ϕ,∇^*p*<0.05, significantly different from the control, AA-injected group, SLZ-treated group, and Dula 50 μg/kg-injected group, respectively, employing Tukey-Kramer multiple comparisons post hoc test after one-way ANOVA

In contrast to control rats, TFF-3 levels were significantly (*p* < 0.05) decreased by 65.02% following intrarectal AA therapy (Fig. 6). The conventional SLZ group demonstrated an important (*p* < 0.05) 2.43-fold boost in colonic TFF-3 levels in contrast to the AA group. Colonic TFF-3 levels raised by 1.07, 1.68, and 2.07 folds, correspondingly, in the Dula-treated groups (50, 100, and 150 μg/kg) contrasted to the AA group. Dula is still more effective at higher dosages (100 and 150 μg/kg) than at lower doses (50 μg/kg).

### Influence of Dula (50, 100, and 150 μg/kg) on colonic expression levels of TGF-β1

**Fig. 7:**
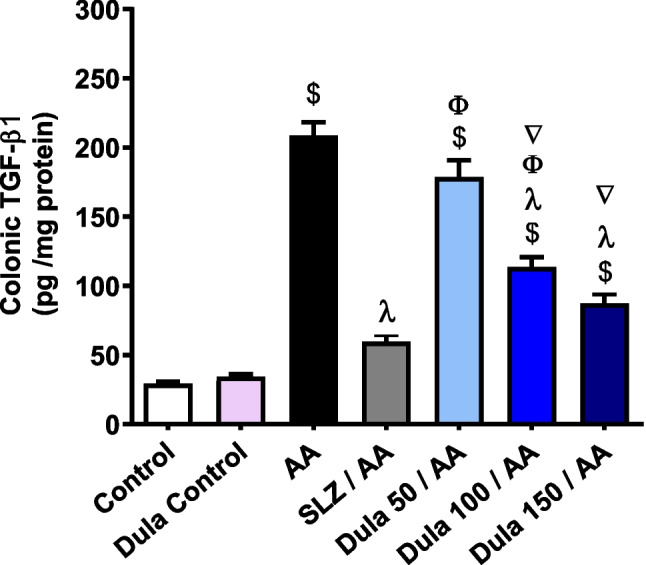
Effect of Dula (50, 100, and 150 μg/kg) on colonic level of TGF-β1. Data were expressed as mean ± SEM (*n* = 8). TGF-β1: transforming growth factor-β; AA: acetic acid; SLZ: sulfasalazine; Dula: dulaglutide. ^$,λ,ϕ,∇^*p*<0.05, significantly different from the control group, AA-injected group, SLZ-treated group, and Dula 50 μg/kg-injected group, respectively, employing Tukey-Kramer multiple comparisons post hoc test after one-way ANOVA

AA inoculation significantly (*p* < 0.05) increased colonic levels of TGF-β1 (Fig. 7) by 7.07-folds, respectively contrasted to normal rats. TGF-β1 levels were decreased meaningly (*p* < 0.05) in the SLZ group contrasted to the AA group, by 71.43%. Nevertheless, in contrast to the AA group, Dula (50 μg/kg) supplementation lowered the rise in TGF-β1 levels by 14.46%, the reduction was not statistically noteworthy. The use of Dula (100 μg/kg) considerably (*p* < 0.05) diminished the high values of TGF-β1 by 45.64%, unlike the group that had AA therapy. The injection of Dula (150 μg/kg) markedly (*p* < 0.05) reduced the excessive levels of the aforementioned parameter by 58.12% versus the AA group.

The results showed no significant differences between the Dula (150 μg/kg) and SLZ groups, showing that the higher dosages of Dula (100 and 150 μg/kg) were more efficient than the lower dosage (50 μg/kg) at lowering the raised levels of TGF-β1.

### Effect of Dula (50, 100, and 150 μg/kg) on colonic levels of PI3K and AKT

**Fig. 8:**
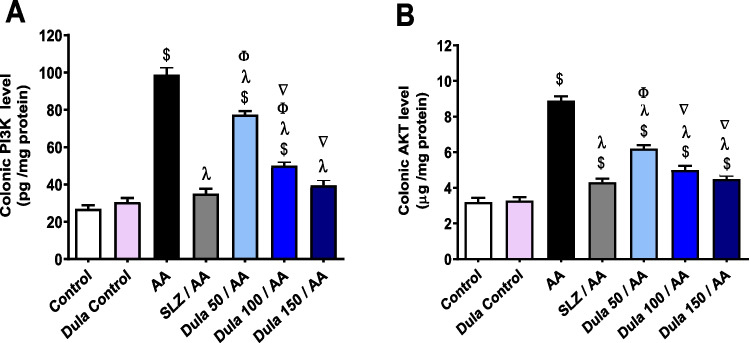
Effect of Dula (50, 100, and 150 μg/kg) on colonic levels of PI3K and AKT. Data were expressed as mean ± SEM (*n* = 8). PI3K: phosphatidylinositol-3-kinase; AKT: protein kinase B; AA: acetic acid; SLZ: sulfasalazine; Dula: dulaglutide. ^$,λ,ϕ,∇^*p*<0.05, significantly different from the control group, AA-injected group, SLZ-treated group, and Dula 50 μg/kg-injected group, respectively, employing Tukey-Kramer multiple comparisons post hoc test after one-way ANOVA

According to Fig. 8, compared to the control group, an intrarectal dose of AA meaningfully (*p* < 0.05) increased colonic levels of PI3K and AKT by 3.67 and 2.77 times, correspondingly. Dula pretreatment (50 μg/kg) ominously (*p* < 0.05) decreased the abnormally high levels of PI3K and AKT by 21.54% and 30.17%, correspondingly, contrasted to the AA group. Dula pretreatment (100 μg/kg) extensively (*p* < 0.05) reduced high levels of PI3K and AKT by 49.16% and 43.87%, correspondingly, in comparison to the AA group. In addition, the pretreatment with Dula (150 μg/kg) meaningfully (*p* < 0.05) diminished these levels by 59.86% and 49.53%, respectively, in comparison to the AA group. Additionally, matched to the AA group, the typical SLZ had a notable (*p* < 0.05) decline in PI3K and AKT levels of 64.40% and 51.41%, respectively.

According to the previous findings, the 150 μg/kg dosage of Dula is superior to lower dosages while not significantly deviating from the SLZ group’s findings in terms of lowering the high levels of PI3K and AKT.

### Efficacy of Dula (50, 100, and 150 μg/kg) on colonic NF-κB and IL-6 expression levels

**Fig. 9:**
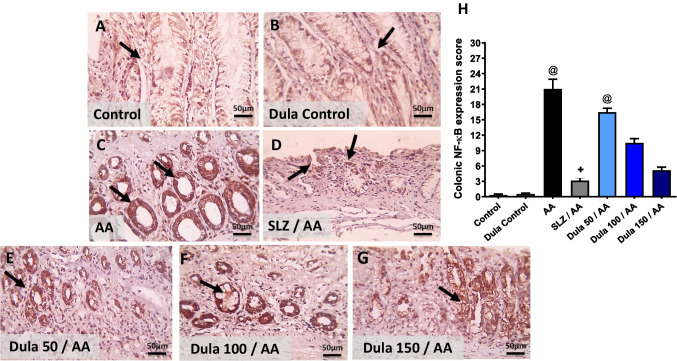
Effect of Dula (50, 100, and 150 μg/kg) on colonic expression level of NF-κB (high magnification X: 400 bar 50. IHC counterstained with Mayer’s hematoxylin). Microscopic pictures of immunostained colonic sections against NF-κB showed the following:** A**, **B** mild positive brown intensity in very few crypts (black arrows) in the control group and Dula control group.** C** a marked increase in positively stained crypts appears (black arrows) in AA-treated group. **D** Mild positive brown staining is seen in a few crypts (black arrows) in SLZ group. **E** Higher numbers of positively stained crypts (black arrows) are seen in Dula (50 μg/kg) more than the SLZ group. **F** Numbers of positively stained crypts (black arrows) are still seen (black arrows) in Dula (100 μg/kg). **G** The positive brown intensity markedly lowered and was observed in a few crypts (black arrows) in Dula (150 μg/kg). **H** NF-κB scores. Data were expressed as median ± IQR. AA: acetic acid; SLZ: sulfasalazine; Dula: dulaglutide; NF-κB: nuclear factor kappa B. ^@,+^*p*<0.05, significantly different from the control and AA-injected groups, respectively, applying the multiple comparison test of Dunn after the Kruskal-Wallis test

Figure 9 illustrates that in the control group and the Dula control group, immune-stained segments against NF-κB displayed light positive brown staining in a very limited number of crypts (Fig. 9A, B). Contrary, the AA group had a noticeable rise in crypts that were positively stained (Fig. 9C). A few crypts had light positive brown staining following the injection of SLZ (Fig. 9D). In comparison to the SLZ group, the Dula (50 μg/kg) group’s colonic sections had more positively stained crypts (Fig. 9E). These increased numbers of positively stained crypts were still seen in the colon segments from Dula (100 μg/kg) group (Fig. 9F). According to colonic sections from the Dula (150 μg/kg) group, the positive brown intensity was only found in a small number of crypts (Fig. 9G). Semi-quantitative analysis of NF-κB expression, as shown in Fig. 9H, proved that contrasted to control rats, animals given AA received substantially higher NF-κB expression scores. In comparison with the AA group, the course therapy with Dula at dosages (100 and 150 μg/kg) and the regular SLZ diminished the NF-κB expression score.

**Fig. 10:**
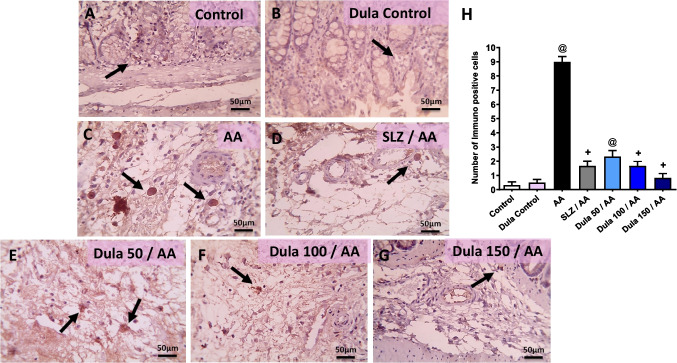
Dula (50, 100, and 150 μg/kg) affects the level of IL-6 expression in the colon (high magnification X: 400 bar 50. IHC counterstained with Mayer’s hematoxylin). Microscopic pictures of immunostained colonic sections against IL-6 showed the following: **A**, **B** no positive cells in submucosa (black arrows) in the control group and Dula control group. **C** Many positive cells appear in submucosa in the AA group. **D** Numbers of positive cells markedly reduced in submucosa (black arrows) in the SLZ-treated group. **E** Few positive cells appear in submucosa (black arrows) in the Dula (50 μg/kg) group. **F** Very few positive cells appear in submucosa (black arrows) in the Dula (100 μg/kg) group. **G** The IL-6 positive cells are accidentally seen in submucosa (black arrows) in the Dula (150 μg/kg) group. **H** IL-6 scores. Data were expressed as median ± IQR. AA: acetic acid; SLZ: sulfasalazine; Dula: dulaglutide. ^@,+^*p*<0.05, significantly different from the control and AA-injected groups, respectively, applying the multiple comparison test of Dunn after the Kruskal-Wallis test

Immune-stained colon slices against IL-6 under a microscope did not exhibit any positive cells in the submucosa in the control group or the Dula control group (Fig. 10A, B). The AA group’s submucosa, on the other hand, had a sizable number of positive cells (Fig. 10C). After SLZ injection, the number of positive cells in the submucosa dramatically reduced (Fig. 10D). There were a few positive cells that appeared in the submucosa in colonic sections from the Dula (50 μg/kg) group (Fig. 10E). Very few positive cells were seen in the submucosa in the Dula (100 μg/kg) group (Fig. 10F). The positive cells are unintentionally discovered in the submucosa of the Dula (150 μg/kg) group (Fig. 10G). In the group that received AA compared to control rodents, there was a substantial rise in the number of immunologically positive cells, as judged by IL-6 release semi-quantitative rating (Fig. 10H). The use of Dula (100 and 150 μg/kg) plus the regular SLZ diminished the quantity of immunologically positive cells in contrast to the AA group.

### IFN-γ colonic expression levels in response to a Dula (50, 100, and 150 μg/kg) treatment

**Fig. 11:**
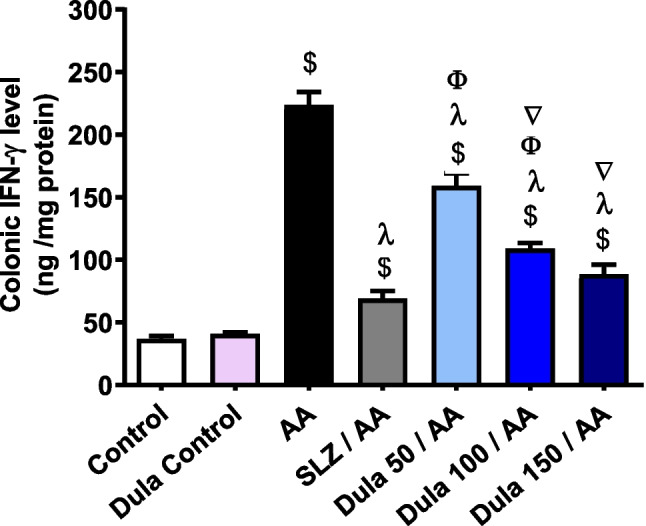
The impact of Dula at 50, 100, and 150 μg/kg on the level of IFN-γ expression in the colon. Data were expressed as mean ± SEM (*n* = 8). IFN-γ: interferon-γ, AA: acetic acid; SLZ: sulfasalazine; Dula: dulaglutide. ^$,λ,ϕ,∇^*p*<0.05, significantly different from the control group, AA-injected group, SLZ-treated group, and Dula 50 μg/kg-injected group, respectively, employing Tukey-Kramer multiple comparisons post hoc test after one-way ANOVA

Figure 11 illustrates that when AA-induced colitis produced a noteworthy (*p* < 0.05) rise in colonic IFN-γ levels by 6.10 folds (*p* < 0.05) compared to the control group. The traditional SLZ revealed an alarming (*p* < 0.05) decline in IFN-γ levels of 69.17% comparable to the AA group. When juxtaposed with the group that got AA therapy, the consumption of the various doses of Dula (50, 100, and 150 μg/kg) considerably (*p* < 0.05) reduced the raised levels of IFN-γ by 28.90%, 51.25%, and 60.45%, correspondingly. Findings indicated that Dula lowered colonic levels of IFN-γ relative to the SLZ group more successfully at high dosages (150 μg/kg) than at low doses (50 and 100 μg/kg).

### Effects of Dula (50, 100, and 150 μg/kg) on serum levels of LDH and CRP

**Fig. 12:**
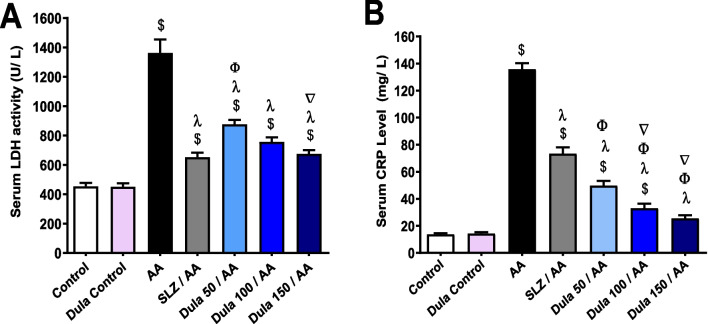
Effect of Dula (50, 100, and 150 μg/kg) on serum levels of LDH and CRP. **A** LDH; **B** CRP. Data were expressed as mean ± SEM (*n* = 8). AA: acetic acid; SLZ: sulfasalazine; Dula: dulaglutide; LDH: lactate dehydrogenase; CRP: C-reactive protein. ^$,λ,ϕ,∇^*p*<0.05, significantly different from the control group, AA-injected group, SLZ-treated group, and Dula 50 μg/kg-injected group, respectively, employing Tukey-Kramer multiple comparisons post hoc test after one-way ANOVA

AA administration meaningfully (*p* <0.05) elevated serum levels of LDH and CRP by 2.99 and 9.88 times, correspondingly, in comparison to control rats (Fig. 12A, B). Following the administration of SLZ, these levels decreased dramatically (*p* < 0.05) by 52.14% and 46%, respectively. In comparison to the AA group, animals delivered Dula (50 μg/kg) had significantly (*p* < 0.05) dropped serum of LDH and CRP (35.59% and 63.39%, respectively). Upon Dula (100 μg/kg) treatment, serum LDH and CRP were considerably lower (44.44% and 75.62%, respectively) (*p* < 0.05) contrary to the AA group.

Dula (150 μg/kg) significantly (*p* < 0.05) decreased blood levels of LDH and CRP by 50.42% and 81.23%, respectively. Notably, Dula (150 μg/kg) reduced serum LDH and CRP more effectively than Dula (50 and 100 μg/kg). CRP levels were significantly lower in the Dula groups compared to the SLZ-treated groups.

## Discussion

The existing investigation proved originally the potential effect of Dula on AA-induced UC. Dula (50, 100, and 150 μg/kg) substantially reduced the colon's weight, the ratio of weight to length, serum CRP, and LDH levels, boosted histopathological alterations and displayed anti-colitis and anti-inflammatory actions. Aside from regulating blood glucose levels, GLP-1, the intestinal hormone, also has anti-inflammatory, anti-apoptotic, and antioxidative effects. Some studies have shown that GLP-1 alleviates macrophage-induced inflammation and relieves the symptoms of UC (Krasner et al. 2014, Anbazhagan et al. 2017b, Hunt et al. 2021), but the underlying mechanism is still not completely understood. In this study, we have shown that s.c. Injection of Dula lowered the effect of tissue injury in AA-induced colitis in rats by enhancing intestinal barrier function *via* increasing TFF-3 and inhibiting intestinal oxidative stress via attenuating MDA and increasing TAC, GSH, and SOD and inflammation *via* regulating TGF-β1, PI3K/AKT, NF-κB, IL-6, and IFN-γ pathways. Our findings suggest that GLP-1 upregulation *via* administering Dula in rats can be considered a potential therapeutic candidate to prevent UC.

### Efficacy of Dula on the colonic expression level of TFF-3: intestinal barrier function

TFF3 enhances intestinal barrier function *via* the regulation of tight junctions to decrease the paracellular permeability of the intestinal epithelium. TFF3 is principally expressed with MUC2 in intestinal goblet cells where it participates in mucosal regeneration and repair (Aihara et al. 2017). Changes in the content of the intestinal mucus may be a factor in IBD’s unbalanced immune response activation (Xavier and Podolsky 2007). It was thought that injury to the epithelium, which mainly impacts goblet cells, produced inflammation (Jang et al. 2022). An earlier study found that the intestinal epithelium of UC patients had lost goblet cells and lacked TFF-3, this exacerbated vulnerability to ongoing inflammation (Aamann et al. 2014). These results aligned with those of Podolsky et al. (Podolsky et al. 2009) who showed that a mouse colitis model generated by dextran sulfate sodium (DSS) had a reduced TFF-3 level. In our study, Dula (50, 100, and 150 μg/kg) groups revealed increased levels of TFF-3 in AA-injected rats proving the promising potential effect of Dula in enhancing intestinal barrier function.

### Effectiveness of Dula on oxidant/antioxidant function: TAC, MDA, GSH, and SOD

Oxidative stress-mediated damage was a key pathophysiological feature of IBD; too much reactive oxygen and nitrogen production was a significant contributor to UC owing to a greater vulnerability to intestinal inflammation and injury brought on by defective normal oxidative defense systems (Mazzolin et al. 2013). Owing to some theories, the positive effects of medications such as corticoids and aminosalicylates, which were employed to treat IBD in individuals, may in part be attributable to their antioxidant properties (Orsi et al. 2014). Oxidative stress was indicated by MDA, as a consequence of the lipid peroxidation process because it was produced excessively when there was an increase in free radicals (Dodda et al. 2014). The natural cell defense system against damage brought on by oxidative stress includes antioxidant enzymes like SOD and GSH, and oxidative stress indicators also include TAC (Cagin et al. 2016). In the existing work, AA-injected group, SOD, GSH, and TAC values were declined, although MDA level was elevated. The findings are in line with other studies that discovered the raised MDA in colons of rats while the levels of GSH and TAC in addition to the enzymatic activity of antioxidants such SOD decreased substantially in two distinct rat models of AA-induced colitis (Cagin et al. 2016, El-Akabawy and El-Sherif 2019a). The oxidative stress state was shown to be reduced after Dula pretreatment (50, 100, and 150 μg/kg), as evidenced by the rise in SOD, TAC, and GSH concentrations and the fall in MDA levels, these results were based to the previous study which concurs with our findings (Wang et al. 2021) where Dula dropped oxidative stress by raising GSH levels and lowering the production of mitochondrial ROS in rat cardiomyocytes damaged by lipopolysaccharide. This was further supported by Li et al.’s work, which indicated that Dula lowered ROS amounts in a model of type II collagen and aggrecan breakdown set on by AGEs in humans (Li et al. 2020). All these findings proved that Dula had a preventive effect against colitis-induced AA that may be posted by its ability to reduce oxidative stress damage.

### Efficacy of Dula on colonic oxidative stress/inflammation: NF-κB, IL-6, and IFN-γ expression levels

Oxidative stress brought on by free radicals is necessary for the transcription factor NF-κB to become activated when it is in its dormant state and combines with IκB (Schreck et al. 1991). Located in the nucleus, the active NF-κB subunits primarily regulate inflammatory genes, making them a crucial regulator of the immune response associated with inflammatory bowel disease (IBD) (Shahid et al. 2022). According to earlier studies, elevated NF-κB levels were linked to higher levels of oxidative stress and inflammatory cytokines, which worsened colon epithelial damage and eventually caused colitis (Melgar et al. 2003, Brenna et al. 2013). Since NF-κB modulated TFF-3 downregulation, Song et al. determined that greater NF-κB expression guided a substantial drop in TFF-3 expression (Song et al. 2006). In our investigation, injection of AA-induced colonic ulceration, a submucosal massive infiltration of chronic inflammatory cells, the creation of lymphoid follicles, gland hyperplasia, and the loss of goblet cells, which decreased the TFF-3 level. In the existing study, AA caused a noteworthy rise in NF-κB, IL-6, and oxidative stress levels in colon tissues that were caused by colitis.

This study identified raised NF-κB levels in the rat colon, which was consistent with the findings of Ala et al. (Ala et al. 2022) who reported that AA enhanced the colon’s NF-κB expression in rats with colitis brought on by AA. In contrast, pretreatment with Dula (50, 100, and 150 μg/kg) reduced the level of NF-κB; our findings were in line with another study (Wang et al. 2021) where Dula therapy produced a substantial reduction in both the activated NF-κB signaling pathway and the level of inflammation, demonstrating a potent inhibitory action of Dula against inflammation in cardiac cells brought on by LPS, and with the study of Ali et al. (Ali et al. 2022) who proved that Dula showed a significant decrease in NF-κB in diabetic rats kidney. The previous study cleared that GLP-1-based administration that had anti-inflammatory consequences on various organs could be utilized to clarify this outcome (Azmy Nabeh et al. 2020). Researchers revealed that Dula’s effects on proinflammatory cytokine expression and ROS generation were mediated via the NF-κB pathway (Li et al. 2020) and that Dula had a strong inhibitory influence on both processes. Also, our investigation revealed that the AA group displayed increased levels of IL-6, which was corroborated by a previous study by Ansari et al. (Ansari et al. 2021) which settled on a Wistar albino rat colitis model generated by AA. The level of IL-6 was dramatically reduced after pretreatment with Dula (50, 100, or 150 μg/kg); this conclusion coincided with the study of Xie et al. (Xie et al. 2022) which was carried out on patients with type 2 diabetes mellitus. Present study establishes that the IFN-γ level was elevated in the AA group, which was consistent with a recent study that found AA-induced colitis in rats substantially elevated the concentration of IFN-γ (El-Akabawy and El-Sherif 2019a). Contrarily, IFN-γ levels were lower after pretreatment with Dula (50, 100, or 150 μg/kg). It was in line with another study that revealed the injection of Dula suppressed the formation of tissue-infiltrating IFN-γ in a mice model of experimental autoimmune encephalomyelitis (Chiou et al. 2019).

### Effect of Dula on colonic levels of PI3K/AKT/TGF-β1 pathway

The PI3K/AKT signal transduction pathway, which had been connected to the emergence and development of UC, regulated and released pro-inflammatory cytokines (Huang et al. 2011) where phosphorylated AKT (p-AKT) activated NF-κB which caused production and release of pro-inflammatory cytokines like TNF-α and IL-1β; this process aided in the production of mucin, an out-of-control cytokine secretion, and other inflammatory responses, resulting in the progression of UC (Gustin et al. 2004, Ibrahim et al. 2020).

Previous investigations revealed a substantial rise in TGF-β protein in the acute and chronic UC groups in contrast to the healthy group of controls (Marafini et al. 2013, Jovanovic et al. 2017, Farghaly 2020). This increase was thought to be a compensatory strategy to relieve the symptoms of UC. TGF-β may therefore be a biomarker for the detection of active UC. The link between TGF-β and PI3K/AKT was discovered via further research because TGF-β has the potential to swiftly trigger PI3K and markedly elevate the phosphorylation of its downstream effector AKT (Zhang 2009, Suwanabol et al. 2012).

The AA group in our research displayed higher levels of TGF-β1/PI3K/AKT and NF-κB as well in comparison to the control group and Dula (50, 100, and 150 μg/kg) groups and, however, showed a noteworthy drop in AKT, PI3K, TGF-β1, and NF-κB levels. These results corroborated the findings of Ali et al.’s study, which showed that TGF-β1 levels dramatically dropped after receiving Dula therapy in male albino rats who had experimentally induced diabetic kidney disease (Ali et al. 2022) and with the previous study of Park et al. which found that Dula decreased TGF-β1 level due to its anti-inflammatory effects on inflammatory signaling, oxidative stress, and cytokine production in obesity-induced airway hyperresponsiveness and fibrosis in a mouse model (Park et al. 2019). Following findings from earlier research (Zaghloul et al. 2022, El Mahdy et al. 2023) which reported raised PI3K and AKT levels in a rat model of AA-induced colitis and a recent study which discovered that acute AA-induced colitis in rats contributed to an upregulation of PI3K and AKT (Xu et al. 2022), this was compatible with our results which showed that the AA group had higher colonic PI3K and AKT levels as compared to the control group. However, Dula (50, 100, and 150 μg/kg) lowered the raised PI3K and AKT levels. To our awareness, this is the original work that confirmed the effect of Dula in decreasing the elevated levels of PI3K and AKT in a rat model of AA-induced colitis.

### Effectiveness of Dula on colonic GLP-1 expression levels

Recent mouse studies had centered on the specific function of GLP-1 in the gastrointestinal system, where it played a significant role in epithelial development but did so via several pathways (Koehler et al. 2015). Additionally, it had been demonstrated that the exogenous injection of GLP-1 analogues protected rats from intestinal damage brought on by chemotherapy (Kissow et al. 2013). In IBD, mucosal repair has become a crucial therapy objective (Neurath and Travis 2012). Increased GLP levels, according to a prior study, improved colitis (Zietek et al. 2017). GLP-1-based therapeutics show anti-inflammatory benefits on numerous organs and systems by lowering the creation of inflammatory cytokines and the proliferation of immune system cells in these tissues (Iwai et al. 2006). These anti-inflammatory effects were thought to be primarily attributable to the GLP-1RA’s capacity to inhibit NF-κB nuclear translocation and phosphorylation; this might trigger the pro-inflammatory cytokines implicated in the origins of IBD to generate fewer of them (Al-Dwairi et al. 2019). In a study by Yusta et al. (Yusta et al. 2015), a GLP-1 receptor knock-out rat model revealed a decline in the production of TGF-1, EGFR, and interleukins like IL-6 which were assumed to be crucial components of the innate immune response. These genes had an impact on mucosal healing and the immune response. Furthermore, it was reported that therapy with GLP-1 enhanced epithelial architecture, lowered the assembly of pro-inflammatory cytokines like TGF, and dramatically delayed the emergence of DSS-induced colitis. Such actions might be a clue to the critical role GLP-1 played in easing the manifestations of IBD (Anbazhagan et al. 2017a).

According to Zatorski et al.’s (Zatorski et al. 2021) study, acute inflammation in mouse models of colitis might cause harm to the colon mucosa and the EEC, which led to the lowering of total GLP-1 levels. Our investigation found that the GLP-1 level dropped in the AA group. As Dula is a structurally modified GLP-1 analogue with a longer half-life (Gupta et al. 2017) and works as a long-acting GLP-1RA, it is unquestionably true that the GLP-1 level in the Dula (50, 100, and 150 μg/kg) groups was elevated. This enhanced epithelial architecture (Zatorski et al. 2019) as a result.

**Fig. 13:**
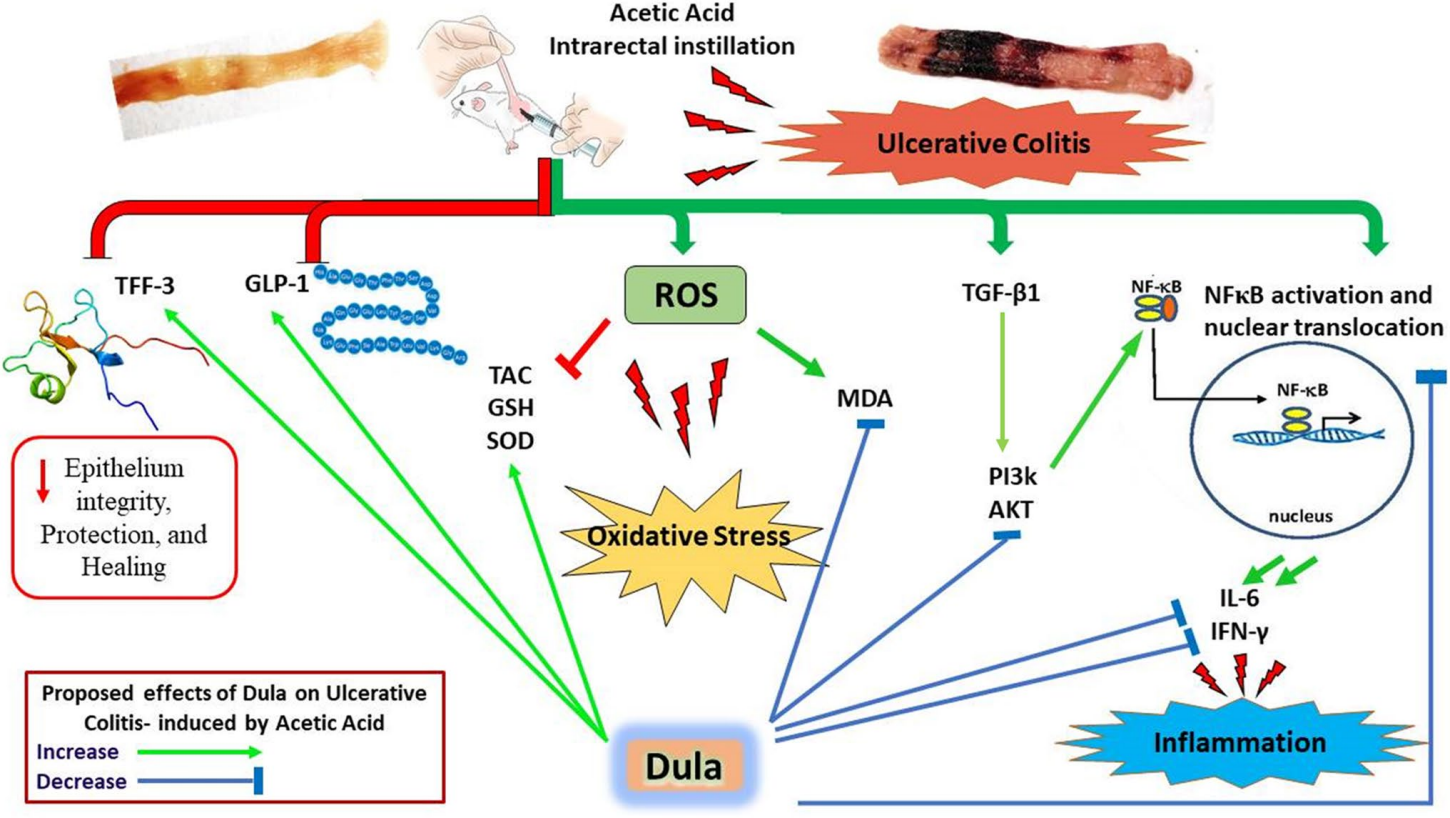
An illustration of the potential cascades that could be involved in Dula’s protective actions against AA-induced UC in rats. TAC is for total antioxidant capacity; Dula stands for dulaglutide; MDA is for malondialdehyde; SOD is for superoxide dismutase; and GSH is for reduced glutathione. Glucagon-like peptide-1 (GLP-1), trefoil Factor-3 (TFF-3), nuclear factor kappa B (NF-κB), interleukin-6 (IL-6), interferon-γ (INF-γ), transforming growth factor-1 (TGF-β1), phosphatidylinositol-3-kinase (PI3K), protein kinase B (AKT), lactate dehydrogenase (LDH), and C-reactive protein (CRP)

Figure 13 displays potential routes that may be connected to Dula’s capability to protect rats against AA-induced colitis.

## Conclusion

Dula, a GLP-1 agonist, may shield rats from AA-induced colitis. Pretreatment with Dula protected against AA-induced UC by decreasing colon mass index, colon weight and weight/length ratio, colonic macroscopic damage, and Dula also showed the ability to reduce oxidative stress damage via decreasing the lipid peroxidation end product, MDA, and restoring the decreased GSH, SOD, and TAC levels. Additionally, Dula managed to downregulate the inflammatory signaling pathway that involves TGF-β1/PI3K/AKT/NF-κB levels in the colonic homogenates and raised the expression of TFF-3 and GLP-1, all of which contribute to a better intestinal barrier. Nevertheless, further studies are needed to assess the safety, tolerability, and clinical effectiveness of Dula in therapy and focus on intestinal mucosal healing.

## Data Availability

All source data for this work (or generated in this study) are available upon reasonable request.
